# Gender-disaggregated longitudinal associations between health behaviours and life satisfaction in community-dwelling older adults: a six-year growth curve analysis of the CLSA

**DOI:** 10.1186/s12966-026-01956-3

**Published:** 2026-07-21

**Authors:** Chiedozie James Alumona, Ogochukwu Kelechi Onyeso, Adesola C. Odole, Laura Vogelsang, Jerome Singleton, Oluwagbohunmi Adetunji Awosoga

**Affiliations:** 1 Faculty of Health Sciences, University of Lethbridge, Lethbridge, AB Canada; 2 Emerging Researchers and Professionals in Ageing-African Network, Abuja, Nigeria; 3 Department of Physiotherapy, College of Basic Medical Sciences, Chrisland University, Abeokuta, Ogun Nigeria; 4 Department of Physiotherapy, Faculty of Clinical Sciences, College of Medicine, University of Ibadan, Ibadan, Oyo Nigeria; 5 Faculty of Health, School of Health and Human Performance, Dalhousie University, Halifax, NS Canada

**Keywords:** CLSA, Well-being, Healthy ageing, Lifestyle, Quality of life

## Abstract

**Background:**

Health behaviours such as physical activity, sedentary behaviour, nutrition, sleep, alcohol consumption, and smoking are modifiable determinants of life satisfaction – an essential outcome for healthy ageing and well-being. We explored the longitudinal association between these health behaviours and life satisfaction among older adults in Canada.

**Methods:**

The study was a secondary analysis of data from the Canadian Longitudinal Study on Aging, focusing on 11,215 adults aged 65 years and older at baseline, with follow-ups 1 and 2 at three-year intervals. Data were analysed using linear mixed-effects growth curve models, stratified by gender, with health behaviours decomposed into within- and between-person components.

**Results:**

At baseline, the participants’ mean (SD) age was 72.94 (5.64) years, and 54.2% were women. Women had lower life satisfaction than men across waves, but the difference was trivial (*B*=-0.24, *p* = 0.035, predicted difference [*pd*]=-0.24 points, effect size [*d*]=-0.04). Men showed a decline in life satisfaction over time, though the effect size was small (*B*=-0.09, *p* = 0.004, *pd*=-0.54 points, *d*=-0.09). Within-person analyses showed that higher alcohol use relative to one’s usual pattern was associated with lower life satisfaction (*B*=-0.23, *p* = 0.037, *pd*=-0.92 points, *d*=-0.15), especially among men, with an appreciable effect size (*B*=-0.35, *p* = 0.024, *pd*=-1.40 points, *d*=-0.24). In contrast, improvements in nutritional risk (*B* = 0.04, *p* < 0.001, *pd* = 0.24 points, *d* = 0.04) and sleep duration (*B* = 0.22, *p* < 0.001, *pd* = 0.32 points, *d* = 0.05) were associated with negligible concurrent increases in life satisfaction. At the between-person level, higher average physical activity across the waves was associated with slightly higher life satisfaction (*B* = 0.01, *p* = 0.004, *pd* = 0.58 points, *d* = 0.10), particularly among men (*B* = 0.01, *p* < 0.001, *pd* = 0.58 points, *d* = 0.10), whereas higher average sedentary time was weakly associated with lower life satisfaction (*B*=-0.04, *p* < 0.001, *pd*=-0.33 points, *d*=-0.06). Lower average nutritional risk (*B* = 0.20, *p* < 0.001, *pd* = 1.22 points, *d* = 0.21) and higher average sleep duration (*B* = 0.35, *p* < 0.001, *pd* = 0.51 points, *d* = 0.09) were associated with higher life satisfaction, with nutrition showing a modest effect size.

**Conclusions:**

The findings demonstrate that behavioural changes and sustained health behaviours are associated with differences in older adults’ life satisfaction, with gender-specific patterns, providing actionable evidence for individuals, healthcare practitioners, and policymakers to promote healthy behaviours.

## Background

Canada has one of the highest life expectancies in the world [1], resulting in a tremendous increase in the older adult population. People aged 65 and above constitute 19% of the population [2], and this proportion is projected to rise to 25% by 2036 [3]. Ensuring that ageing is accompanied by sustained well-being has become a national priority, as reflected in the mandate of the Canadian Institutes of Health Research (CIHR) Institute of Aging, which supports research aimed at promoting healthy ageing and enhancing quality of life across the later life course [4].

Life satisfaction is a core component of well-being and is defined as a global cognitive evaluation of one’s overall quality of life [5]. It is a subjective judgement that reflects the difference between an individual’s current situation and their perceived ideal life standard [5]. In later life, higher life satisfaction has been associated with greater social engagement, reduced disability, and lower mortality risk [6, 7]. As such, it has become a crucial outcome in healthy ageing, consistent with the WHO’s *Healthy Ageing Framework*, which emphasises maintaining functional ability and well-being across the life course rather than focusing solely on the absence of disease [8].

The determinants of life satisfaction in later life have been broadly categorised into demographic and socioeconomic factors, health status, social relationships, and health behaviours [9, 10]. Among these, health behaviours such as physical activity, sedentary behaviour, nutrition, sleep, alcohol consumption, and smoking are crucial because they are dynamic, modifiable, and responsive to both individual-level choices and policy-level interventions [11]. These behaviours serve as mechanisms through which older adults adapt to age-related changes, thereby promoting life satisfaction and healthy ageing [8]. Physical activity, for instance, helps preserve functional capacity, enabling older adults to maintain independence in daily activities and thereby enhancing life satisfaction through sustained control over their lives.

Despite their importance, most quantitative studies examining associations between health behaviours and life satisfaction have relied on cross-sectional designs [9, 10, 12–14]. While these studies provide valuable snapshots, they do not capture the dynamic nature of health behaviours and life satisfaction, nor distinguish stable individual differences from within-individual changes over time. Consequently, there is a knowledge gap on how changes in health behaviours are associated with life satisfaction trajectories in later life.

In addition to these methodological limitations, the potential role of gender in shaping these associations remains underexplored, despite evidence that health behaviours are socially patterned by gender roles, expectations, and norms across the life course [15]. Gender shapes exposure to stressors, distribution of caregiving and work responsibilities, and access to resources (e.g., income, social support), all of which enable or constrain engagement in health behaviours [16–19]. In addition, gender norms surrounding masculinity and femininity may differentially encourage or discourage behaviours such as help-seeking, physical activity, and substance use, leading to distinct behavioural patterns across gender groups [20]. These social patterns suggest that the association between health behaviours and life satisfaction may differ based on gender; however, few studies have examined these differences [9, 21]. Therefore, there is a need for longitudinal, population-based, gender disaggregated evidence on the associations between health behaviours and life satisfaction.

The present study addresses these gaps by using data from the Canadian Longitudinal Study on Aging (CLSA), which assessed life satisfaction and health behaviours at baseline, first follow-up, and second follow-up, culminating in a six-year follow-up. Using a longitudinal analytical approach, this study examined how changes in health behaviours within individuals and between individuals are associated with life satisfaction over time among Canadian adults aged 65 years and older. Additionally, we completed gender-disaggregated models to assess whether these longitudinal associations differ for men and women. We also examined the cross-sectional bivariate associations between each health behaviour and life satisfaction at baseline and follow-ups. The study outcomes provide evidence for individuals, healthcare workers, and policymakers to encourage health-promoting behaviours and foster healthy ageing.

## Methods

### Study design and data source

The study was a secondary longitudinal analysis of the CLSA data. The CLSA is an ongoing national longitudinal study that commenced in 2011 with 51,338 individuals aged 45 to 85 years (comprehensive cohort = 30,097; tracking cohort = 21,241) who have been followed up every three years [22, 23]. The CLSA excluded participants who were full-time members of the Canadian Armed Forces, lived in long-term care homes at baseline, lived on Federal First Nations reserves or settlements, lived in the three territories or some remote regions, had cognitive impairment at the time of recruitment, and were unable to communicate in English or French [22]. The CLSA’s study protocol, procedures, consent, and ethics approval have been extensively discussed in previous publications [22, 23]. More information about the CLSA is available at www.clsa-elcv.ca. We analysed data from the comprehensive cohort because they were collected in person and included sleep measures [22]. The analytic sample comprised 11,215 participants aged 65 years or older at baseline who completed assessments at 3-year intervals. The study was approved by the Human Research Ethics Board of the University of Alberta (reference number: Pro00149975).

### Outcome variable

The outcome variable, life satisfaction, was measured using the Satisfaction with Life Scale (SWLS) [5]. The SWLS is a 5-item questionnaire that assesses people’s global life satisfaction without recourse to any specific life domains. It is a 7-point Likert scale, with one and seven representing “strongly disagree” and “strongly agree,” respectively. The possible total score ranges from 5 to 35, with higher scores indicating greater life satisfaction. The included participants completed all items on the scale. The scale is valid and reliable [5, 24] and has been translated into more than 30 languages, with satisfactory psychometric properties, indicating its wide utility [24]. The SWLS has also demonstrated good psychometric properties among older adults [25] and is sensitive to detecting changes in life satisfaction following major life events and transitions [24].

### Predictors

#### Smoking, alcohol consumption and sleep

Participants indicated their smoking status by choosing one of the three responses: yes (I currently smoke), former (I don’t smoke now, but I have in the past), or no (I don’t smoke, and I never have). Frequency of alcohol consumption over the past 12 months was assessed with options as never, less than once a month, once a month, 2–3 times a month, once a week, 2–3 times a week, 4–5 times a week, and almost daily. Sleep was measured as the self-reported average number of hours of actual sleep at night over the past month.

#### Physical activity

Physical activity level was obtained using the Physical Activity Scale for the Elderly questionnaire [26]. The questionnaire assesses the frequency of engagement in recreational activities (light, moderate, and strenuous), walking, and muscle-strengthening/endurance exercises over the past seven days, as well as the average daily duration of these activities. The remaining items assessed participation in house chores, home care, caring for another person, and work or volunteer activities in the past seven days. The total score was computed by multiplying either the time spent in each activity (hours per day) or the participation (i.e., yes/no) in each activity by empirically derived item weights and then summing all of them [27]. The scale is valid and reliable and has been translated into several languages, with acceptable psychometric properties [28].

#### Sedentary time

Participants were asked how frequently they sat over the past seven days for activities such as reading, watching TV, using a computer, and making handicrafts, as well as the average daily duration of these activities. The response options for frequency were never (0 days), seldom (1–2 days), sometimes (3–4 days), and often (5–7 days). The answer options for the duration were less than one hour, one hour but less than two hours, two hours but less than four hours, and four hours or more. The weekly sedentary time was estimated by multiplying the midpoint of the frequency and duration category, except for the ≥ 4-hour category, which was coded as 5 h [29, 30]. For example, if a participant reported 3–4 days and 2–4 h of sitting activities, the value would be 10.5 h per week (3.5 days x 3 h).

#### Nutritional risk

Nutritional risk was assessed using the Abbreviated Seniors in the Community Risk Evaluation for Eating and Nutrition II (AB SCREEN™ II) [31]. The tool comprises eight questions on weight change, skipping meals, appetite, swallowing, fruit and vegetable servings, fluid intake, eating with others, and meal preparation. The total score ranges from 0 to 48, with lower scores representing higher nutritional risk. The AB SCREEN™ II is a valid and reliable tool for identifying the risk of impaired nutritional states in community-living older adults [31]. The AB SCREEN™ II assessment tool is owned by Dr Heather Keller. Use of the AB SCREEN™ II assessment tool in the CLSA was made under license from the University of Guelph.

#### Covariates

The multivariate models were controlled for covariates, such as race, age, gender, marital status, educational attainment, wealth, self-rated health, and social support [9, 10, 12]. Social support was measured using the 19-item Medical Outcomes Study Social Support Survey, with a transformed score ranging from 0 to 100 and higher scores indicating higher social support [32].

### Variable description

Continuous variables: life satisfaction score, physical activity score, sedentary time (hours per week), nutritional risk score, sleep (average hours per night), age (years), and social support score. Category variables: smoking status, (never smoked, former, current smoker), gender (men, women), marital status (has no partner, has partner), self-rated health (poor, good), wealth (less than $50,000, $50,000 to <$100,000, $100,000 to <$1 M, $1 M and above), education (secondary or less, above secondary), education gain (no, yes), religious affiliation (no, yes), race (non-Caucasian, Caucasian). Alcohol frequency (never, less than once a month, once a month, 2–3 times a month, once a week, 2–3 times a week, 4–5 times a week, and almost daily) was treated as an ordinal continuous variable.

### Data analysis

Data were analysed using the Statistical Package for the Social Sciences (SPSS) version 31 and R version 4.3.2, incorporating the dplyr, tidyr, tidyverse, lme4, lmerTest, and broom.mixed packages. All analyses were completed by applying the CLSA inflation weight for descriptives and the analytic weight for inferential statistics. Participants’ characteristics were summarised using descriptive statistics: frequencies, percentages, means, and standard deviations.

Before the inferential analysis, continuous variables were tested for normality, and multicollinearity was assessed using the variance inflation factor (VIF) < 4 [33]. Consistent with population-based research, life satisfaction scores were negatively skewed, showing that a larger proportion of participants reported high satisfaction than low and moderate [34]. However, the raw score was still used because the residual diagnostics from the linear mixed-effects growth curve models were approximately normal, and the interpretation was clearer [9].

Comparisons of participants’ characteristics between men and women were conducted using chi-square tests for categorical variables and independent-samples t-tests for continuous variables in each wave. Bivariate regressions were completed for the full sample and separately by gender to estimate zero-order associations between the outcome and each predictor variable at each wave.

Longitudinal associations between health behaviours and life satisfaction were examined using linear mixed-effects growth curve models in R software, with repeated observations nested within individuals. Growth curve analysis was employed instead of traditional multivariate regression to avoid listwise deletion of participants with incomplete data [35]. Life satisfaction was modelled as a function of time, health behaviours, and covariates. All variables were time-varying, except for gender, religious affiliation, race, and education, which were measured at baseline and treated as time-invariant covariates. Health behaviours were decomposed into within-person and between-person components. The within-person components captured deviations from an individual’s average behaviour over time (i.e., behaviour at a given wave minus the individual’s mean across waves), whereas the between-person components represented stable differences between individuals across the study period (i.e., person’s mean level across waves). A fully adjusted growth-curve model was first estimated in the full sample and subsequently stratified by gender to examine potential gender differences. Model fit was assessed using the log-likelihood, Akaike Information Criterion, and Bayesian Information Criterion. The alpha level was set at 0.05 for all the inferential statistics.

Predicted differences for meaningful exposure contrasts and effect sizes were computed for all predictors to address potential inflation of statistical significance attributable to a large sample size. For each continuous predictor, the predicted difference (*pd*) in life satisfaction was calculated by multiplying the unstandardized regression coefficient (*B*) by one standard deviation (SD) of that predictor [36]. This approach yields an estimate of the expected change in SWLS score associated with a clinically and practically meaningful shift in each exposure. For alcohol frequency, which was measured on an eight-level ordinal scale (0 = never to 7 = almost daily), the meaningful contrast was defined as a four-level increase corresponding to a shift from never drinking to drinking once per week. The contrast for “time” spanned the full six-year follow-up period (0 to 6 years). For binary and categorical predictors, the contrast was defined as the difference between the reference category and the comparison group. Standardised effect sizes were then calculated as Cohen’s d, dividing each predicted difference by the SD of the outcome (SWLS), and interpreted using conventional benchmarks of small (*d* = 0.20), medium (*d* = 0.50), and large (*d* = 0.80) [37].

## Results

### Participant’s characteristics

A total of 11,215 participants were included in the analyses. At baseline, the participants’ mean (SD) age was 72.94 (5.64) years, with 54.2% identifying as women. The majority were Caucasians (96.1%) and had an education level above secondary (56.9%). Table 1 shows the gender differences in the distribution of participants’ characteristics. A higher proportion of men than women were current smokers (Baseline: ꭓ² = 24502.112, *p* < 0.001) and daily drinkers (Baseline: ꭓ² = 37360.972, *p* < 0.001) and these results were consistent across follow-ups 1 and 2. Similarly, Table 2 shows that men have a higher life satisfaction (Baseline: *t* = 102.902, *p* < 0.001), physical activity (Baseline: *t* = 159.786, *p* < 0.001), sleep hours per night (Baseline: *t* = 47.865, *p* < 0.001), and lower nutritional risk (Baseline: *t* = 66.392, *p* < 0.001) than women. In contrast, women have higher sedentary time (Baseline: *t* = -11.701, *p* < 0.001) than men. These findings were replicated at both follow-up assessments (Tables 1 and 2).

**Table 1:** Participants’ characteristics at baseline and follow-ups (*n* = 11,215)

Variable	Baseline					Follow-up 1					Follow-up 2				
	Percentage (%)			ꭓ2-statistic	*p*-value	Percentage (%)			ꭓ2-statistic	*p*-value	Percentage (%)			ꭓ2-statistic	*p*-value
	Total	Men	Women			Total	Men	Women			Total	Men	Women		
Smoking status				24502.112	< 0.001*				20977.502	< 0.001*				14710.944	< 0.001*
Never smoked	39.7	31.5	46.7			43.3	34.9	50.4			47.1	39.4	53.5		
Former smoker	53.5	60.8	47.3			51.6	58.8	45.5			49.1	55.9	43.5		
Current smoker	6.8	7.8	6.0			5.1	6.3	4.1			3.8	4.7	3.0		
Alcohol frequency				37360.972	< 0.001*				30360.441	< 0.001*				23448.150	< 0.001*
Never	16.4	13.2	19.1			13.3	11.6	14.7			19.0	15.7	21.7		
Less than once a month	13.7	9.2	17.6			15.4	10.5	19.6			13.9	10.5	16.7		
Once a month	6.7	5.8	7.5			7.5	6.8	8.1			8.1	7.08	8.3		
2–3 times a month	8.2	7.5	8.8			8.4	8.1	8.7			8.1	7.1	8.9		
Once a week	9.6	9.8	9.5			10.5	10.3	10.7			9.2	8.5	9.7		
2–3 times a week	15.8	18.1	13.7			15.4	15.5	15.4			14.6	16.0	13.5		
4–5 times a week	6.9	7.4	6.5			7.1	7.8	6.5			7.1	8.1	6.2		
Almost daily	22.6	28.9	17.2			22.3	29.4	16.2			20.1	26.3	15.0		
Marital status				67986.163	< 0.001*				72975.023	< 0.001*				75179.364	< 0.001*
Has no partner	33.9	20.6	45.2			35.7	20.6	48.3			38.1	21.2	52.0		
Has partner	66.1	79.4	54.8			64.3	79.4	51.7			61.9	78.8	48.0		
Self-rated health				72.146	< 0.001*				52.398	< 0.001*				23.079	< 0.001*
Poor	10.0	9.7	10.0			11.7	11.4	11.9			13.9	14.1	13.7		
Good	90.0	90.3	89.8			88.3	88.6	88.1			86.1	85.9	86.3		
Wealth (savings and investments)				35006.456	< 0.001*				24803.067	< 0.001*				27075.251	< 0.001*
Less than $50,000	27.8	19.6	35.6			27.6	20.7	33.8			27.7	20.0	34.5		
$50,000 - <$100,000	16.9	15.5	18.3			17.9	16.0	19.6			15.9	14.3	17.4		
$100,000 - <$1 M	45.9	52.1	40.0			45.1	50.8	39.8			45.6	50.5	41.3		
$1 M and above	9.4	12.8	6.1			9.5	12.5	6.7			10.7	15.2	6.8		
Religious affiliation				11272.241	< 0.001*										
No	17.6	22.0	13.9												
Yes	82.4	78.0	86.1												
Education gain									1404.487	< 0.001*				381.743	< 0.001*
No						96.0	96.9	95.3			98.3	98.6	98.0		
Yes						4.0	3.1	4.7			1.7	1.4	2.0		

Source: weighted Canadian Longitudinal Study on Ageing dataset

* = Chi-square is significant at *p* < 0.05. *N* ≠ 11,215 in variables with missing data. The percentage was calculated with valid cases

**Table 2:** Participants’ characteristics (continuous variables) at baseline and follow-ups

Variable	Baseline				
	Mean ± SD			t-statistic	*p*-value
	Total	Men	Women		
Life satisfaction score	28.28 **±** 5.94	28.94 ± 5.70	27.72 ± 6.08	102.902	< 0.001*
Physical activity score	114.36 ± 57.78	124.75 ± 62.86	105.59 ± 51.53	159.786	< 0.001*
Sedentary time (hours/week)	21.68 ± 8.24	21.57 ± 8.30	21.77 ± 8.18	-11.701	< 0.001*
Nutritional risk score	38.80 ± 6.11	39.25 ± 5.78	38.41 ± 6.35	66.392	< 0.001*
Sleep (hours/night)	6.91 ± 1.46	6.99 ± 1.31	6.85 ± 1.57	47.865	< 0.001*
Age (years)	72.94 ± 5.64	72.83 ± 5.61	73.04 ± 5.67	-18.810	< 0.001*
Social support score	79.13 ± 18.11	80.37 ± 18.26	78.08 ± 17.91	62.605	< 0.001*
	Follow-up 1				
Life satisfaction score	28.72 ± 5.83	29.17 ± 5.56	28.33 ± 6.01	66.914	< 0.001*
Physical activity score	105.54 ± 53.20	114.37 ± 57.11	98.13 ± 48.46	141.825	< 0.001*
Sedentary time (hours/week)	24.04 ± 7.76	24.00 ± 7.67	24.08 ± 7.83	-4.904	< 0.001*
Nutritional risk score	37.05 ± 8.58	37.94 ± 8.00	36.29 ± 8.98	89.968	< 0.001*
Sleep (hours/night)	6.88 ± 1.42	6.98 ± 1.36	6.78 ± 1.47	64.857	< 0.001*
Age (years)	75.56 ± 5.53	75.40 ± 5.48	75.70 ± 5.57	-25.392	< 0.001*
Social support score	79.23 ± 18.32	80.17 ± 19.09	78.41 ± 17.59	42.886	< 0.001*
	Follow-up 2				
Life satisfaction score	28.76 ± 5.80	29.30 ± 5.59	28.32 ± 5.93	72.935	< 0.001*
Physical activity score	100.78 ± 50.53	107.78 ± 54.55	95.03 ± 46.18	107.749	< 0.001*
Sedentary time (hours/week)	25.01 + 7.21	24.92 ± 7.17	25.09 ± 7.25	-10.343	< 0.001*
Nutritional risk score	37.52 ± 6.50	38.71 ± 5.90	36.55 ± 6.81	144.401	< 0.001*
Sleep (hours/night)	6.99 ± 1.40	7.11 ± 1.34	6.89 ± 1.45	68.564	< 0.001*
Age (years)	77.93 ± 5.39	77.83 ± 5.41	78.02 ± 5.37	-15.095	< 0.001*
Social support score	81.27 ± 17.73	82.39 ± 18.22	80.33 ± 17.25	47.914	< 0.001*

Source: weighted Canadian Longitudinal Study on Ageing dataset

* = Independent samples t-test is significant at *p* < 0.05

### Bivariate analysis

There was a significant zero-order association between life satisfaction and health behaviours, such that greater physical activity (Baseline: *β* = 0.14, *p* < 0.001), lower nutritional risk (Baseline: *β* = 0.31, *p* < 0.001), longer sleep duration (Baseline: *β* = 0.16 *p* < 0.001), higher alcohol frequency (Baseline: *β* = 0.13 *p* < 0.001), and lower sedentary time (Baseline: *β* = -0.09 *p* < 0.001) were associated with higher life satisfaction in the “both gender” model (Table 3). These associations were statistically significant and directionally consistent at both follow-up assessments and within gender-stratified analyses at each wave. Being a non-smoker (Baseline: *β* = 0.06, *p* < 0.001) and a current smoker (Baseline: *β* = -0.08, *p* < 0.001) were positively and negatively associated with higher life satisfaction, respectively. Similar results were also observed at both follow-up waves and in the men and women models at each wave (Table 3).

**Table 3:** Bivariate regression (zero-order association) between health behaviours and life satisfaction at baseline and follow-ups

Variable	Baseline					
	Both Gender		Men		Women	
	β-statistic	*p*-value	β-statistic	*p*-value	β-statistic	*p*-value
Alcohol frequency (unit increase)	0.13	< 0.001*	0.10	< 0.001*	0.12	< 0.001*
Physical activity (unit increase)	0.14	< 0.001*	0.12	< 0.001*	0.13	< 0.001*
Sedentary time (increase in hours/week)	-0.09	< 0.001*	-0.09	< 0.001*	-0.08	< 0.001*
Nutritional risk score (unit increase)	0.31	< 0.001*	0.29	< 0.001*	0.32	< 0.001*
Sleep (increase in hours/night)	0.16	< 0.001*	0.18	< 0.001*	0.14	< 0.001*
Smoking status						
Never smoked	0.06	< 0.001*	0.06	< 0.001*	0.08	< 0.001*
Former smoker	-0.01	0.212	-0.02	0.305	-0.04	0.017*
Current smoker	-0.08	< 0.001*	-0.07	< 0.001*	-0.11	< 0.001*
	Follow-up 1					
Alcohol frequency (unit increase)	0.11	< 0.001*	0.07	< 0.001*	0.13	< 0.001*
Physical activity (unit increase)	0.16	< 0.001*	0.18	< 0.001*	0.13	< 0.001*
Sedentary time (increase in hours/week)	-0.09	< 0.001*	-0.10	< 0.001*	-0.09	< 0.001*
Nutritional risk (unit increase)	0.28	< 0.001*	0.29	< 0.001*	0.25	< 0.001*
Sleep (increase in hours/night)	0.13	< 0.001*	0.14	< 0.001*	0.12	< 0.001*
Smoking status						
Never smoked	0.07	< 0.001*	0.08	< 0.001*	0.09	< 0.001*
Former smoker	-0.03	0.016*	-0.04	0.026*	-0.04	0.014*
Current smoker	-0.08	< 0.001*	-0.07	< 0.001*	-0.11	< 0.001*
	Follow-up 2					
Alcohol frequency (unit increase)	0.09	< 0.001*	0.08	< 0.001*	0.08	< 0.001*
Physical activity (unit increase)	0.15	< 0.001*	0.15	< 0.001*	0.13	< 0.001*
Sedentary time (increase in hours/week)	-0.07	< 0.001*	-0.06	0.002*	-0.07	< 0.001*
Nutritional risk (unit increase)	0.28	< 0.001*	0.28	< 0.001*	0.28	< 0.001*
Sleep (increase in hours/night)	0.10	< 0.001*	0.08	< 0.001*	0.11	< 0.001*
Smoking status						
Never smoked	0.07	< 0.001*	0.08	< 0.001*	0.09	< 0.001*
Former smoker	-0.03	0.014*	-0.04	0.028*	-0.04	0.015*
Current smoker	-0.09	< 0.001*	-0.07	< 0.001*	-0.14	< 0.001*

Source: weighted Canadian Longitudinal Study on Ageing dataset. Standardised regression coefficient (β) = zero-order correlation coefficient (r)

* = β was significant at *p* < 0.05

### Multivariate longitudinal analysis

Table 4 shows that the baseline life satisfaction was significantly above zero in the “both gender” model (intercept: *B* = 9.47, *p* < 0.001), men (*B* = 8.71, *p* < 0.001), and women (*B* = 10.11, *p* < 0.001), indicating that participants’ baseline life satisfaction was moderate-to-high. Although women had statistically significant lower life satisfaction than men across waves (gender: *B* = -0.24, *p* = 0.035), the predicted difference (*pd* = -0.24 SWLS points) and effect size (*d* = -0.04) were of trivial magnitude. In the “both gender” model, life satisfaction showed a statistically significant decline over time (time: *B* = -0.07, *p* < 0.001); however, the estimated six-year change was negligible (*pd* = -0.42 points, *d* = -0.07). A similar pattern was observed among men, where the decline was statistically significant (*B* = -0.09, *p* = 0.004), but the effect size was small (*pd* = -0.54 points, *d* = -0.09).

**Table 4:** Linear growth curve: Fixed effects of time and health behaviours on life satisfaction

Variable	Both Gender				Men				Women			
	B-statistic	95% CI		*p*-value	B-statistic	95% CI		*p*-value	B-statistic	95% CI		*p*-value
		Lower	Upper			Lower	Upper			Lower	Upper	
Intercept	9.47	7.39	11.55	< 0.001*	8.71	5.89	11.53	< 0.001*	10.11	7.07	13.15	< 0.001*
Time	-0.07	-0.11	-0.03	< 0.001*	-0.09	-0.15	-0.03	0.004*	-0.06	-0.12	0.00	0.098
*Within-person change*												
Alcohol frequency (level change)	-0.23	-0.45	-0.01	0.037*	-0.35	-0.66	-0.04	0.024*	-0.15	-0.44	0.14	0.324
Physical activity (unit change)	0.00	0.00	0.00	0.930	0.00	0.00	0.00	0.902	0.00	0.00	0.00	0.773
Sedentary time (unit change)	0.00	-0.02	0.02	0.984	0.00	-0.02	0.02	0.785	0.00	-0.02	0.02	0.864
Nutritional risk score (unit change)	0.04	0.02	0.06	< 0.001*	0.04	0.02	0.06	0.003*	0.04	0.02	0.06	0.008*
Sleep (unit change)	0.22	0.12	0.32	< 0.001*	0.16	0.02	50.30	0.021*	0.26	0.12	0.40	< 0.001*
*Between-person difference*												
Alcohol frequency (higher level)	0.10	-0.06	0.26	0.237	-0.10	-0.34	0.14	0.397	0.26	0.02	0.50	0.025
Physical activity (unit higher)	0.01	-0.01	0.03	0.004*	0.01	0.01	0.01	< 0.001*	0.00	0.00	0.00	0.775
Sedentary time (unit higher)	-0.04	-0.06	-0.02	< 0.001*	-0.05	-0.07	-0.03	< 0.001*	-0.04	-0.06	-0.02	0.006*
Nutritional risk score (unit higher)	0.20	0.18	0.22	< 0.001*	0.20	0.16	0.24	< 0.001*	0.21	0.17	0.25	< 0.001*
Sleep (unit higher)	0.35	0.25	0.45	< 0.001*	0.45	0.31	0.59	< 0.001*	0.28	0.16	0.40	< 0.001*
† Smoking status (ref: never)												
Former smoker	-0.07	-0.27	0.13	0.480	0.04	-0.23	0.31	0.754	-0.22	-0.51	0.07	0.134
Current smoker	-0.43	-0.92	0.06	0.079	-0.47	-1.08	0.14	0.129	-0.38	-1.16	0.40	0.353
*Covariates*												
Gender (ref: men)	-0.24	-0.48	0.00	0.035*	-			-	-			-
Age (increase in years)	0.01	-0.01	0.03	0.313	0.02	0.00	0.04	0.144	-0.01	-0.05	0.03	0.728
Marital status (ref: has no partner)	-0.41	-0.65	-0.17	< 0.001*	-0.13	-0.48	0.22	0.479	-0.58	-0.89	-0.27	< 0.001*
Self-rated health (ref: poor)	3.06	2.81	3.31	< 0.001*	2.99	2.64	3.34	< 0.001*	3.16	2.77	3.55	< 0.001*
Wealth (savings and investments) (ref: Less than $50,000)												
$50,000 - <$99,999	0.35	0.10	0.60	0.008*	0.21	-0.16	0.58	0.270	0.42	0.07	0.77	0.021*
$100,000 - <$999,99	0.53	0.29	0.77	< 0.001*	0.67	0.34	1.00	< 0.001*	0.40	0.07	0.73	0.020*
$1 M and above	0.92	0.57	1.27	< 0.001*	1.05	0.60	1.50	< 0.001*	0.79	0.20	1.38	0.008*
Social support score (increase in score)	0.07	0.05	0.09	< 0.001*	0.06	0.04	0.08	< 0.001*	0.08	0.06	0.10	< 0.001*
Race (non-Caucasian)	0.43	-0.20	1.06	0.177	0.13	-0.63	0.89	0.737	0.91	-0.13	1.95	0.086
Religious affiliation (ref: yes)	0.72	0.45	0.99	< 0.001*	0.95	0.62	1.28	< 0.001*	0.39	-0.04	0.82	0.072
Education (ref: Secondary or less)	-0.14	-0.39	0.11	0.293	-0.17	-0.54	0.20	0.350	-0.10	-0.45	0.25	0.566
Education gain (ref: no)	-0.31	-0.74	0.12	0.149	-0.16	-0.75	0.43	0.593	-0.42	-1.03	0.19	0.167
Model fit indices												
Log-likelihood	-42378.7				-22133.2				-20190.7			
Akaike’s Information Criterion (AIC)	84815.3				44322.5				40437.4			
Schwarz’s Bayesian Criterion (BIC)	85034.2				44515.9				40627.8			

Source: weighted Canadian Longitudinal Study on Ageing dataset

*CI* Confidence interval

* = B was significant at *p* < 0.05. † = non-decomposed predictor

Within-person analyses showed that moving to a higher level of alcohol use relative to one’s usual pattern was associated with lower life satisfaction in the “both gender” model (*B* = -0.23, *p* = 0.037, *pd* = -0.92 points, *d* = -0.15). This association was driven primarily by men, with an appreciable effect size (*B* = -0.35, *p* = 0.024, *pd* = -1.40 points, *d* = -0.24). In contrast, improvements in nutritional risk and sleep duration were consistently associated with negligible concurrent increases in life satisfaction in the “both gender” model (nutrition: *B* = 0.04, *p* < 0.001, *pd* = 0.24 points, *d* = 0.04; sleep: *B* = 0.22, *p* < 0.001, *pd* = 0.32 points, *d* = 0.05), and in both the men (nutrition: *B* = 0.04, *p* = 0.003, *pd* = 0.24 points, *d* = 0.04; sleep: *B* = 0.16, *p* = 0.021, *pd* = 0.23 points, *d* = 0.04) and women (nutrition: *B* = 0.04, *p* = 0.008, *pd* = 0.24 points, *d* = 0.04; sleep: *B* = 0.26, *p* < 0.001, *pd* = 0.38 points, *d* = 0.06) models.

At the between-person level, individuals with higher average physical activity across the three waves had a slightly greater life satisfaction in the “both gender” model (*B* = 0.01, *p* = 0.004, *pd* = 0.58 points, *d* = 0.10) and men’s model (*B* = 0.01, *p* < 0.001, *pd* = 0.58 points, *d* = 0.10). Conversely, higher average sedentary time across waves was weakly associated with lower life satisfaction in the “both gender” model (*B* = -0.04, *p* < 0.001, *pd* = -0.33 points, *d* = -0.06) and in both gender-disaggregated models (men: *B* = -0.05, *p* < 0.001, *pd* = -0.41 points, *d* = -0.07; women: *B* = -0.04, *p* = 0.006, *pd* = -0.33 points, *d* = -0.06). Lower average nutritional risk and higher average sleep duration were associated with higher life satisfaction in the “both gender” sample, with nutrition showing a modest effect size (nutrition: *B* = 0.20, *p* < 0.001, *pd* = 1.22 points, *d* = 0.21; sleep: *B* = 0.35, *p* < 0.001, *pd* = 0.51 points, *d* = 0.09). Similar associations were observed in both men (nutrition: *B* = 0.20, *p* < 0.001, *pd* = 1.22 points, *d* = 0.21; sleep: *B* = 0.45, *p* < 0.001, *pd* = 0.66 points, *d* = 0.11) and women (nutrition: *B* = 0.21, *p* < 0.001, *pd* = 1.28 points, *d* = 0.22; sleep: *B* = 0.28, *p* < 0.001, *pd* = 0.41 points, *d* = 0.07).

## Discussion

Life satisfaction, which has become a central focus for stakeholders seeking to promote healthy ageing, is shaped by modifiable health behaviours, such as physical activity, sedentary behaviour, nutrition, sleep, alcohol consumption, and smoking [38]. Therefore, we explored how changes in these health behaviours relate to changes in life satisfaction over time. Overall, life satisfaction declined marginally over time, after controlling for health behaviours, sociodemographic, health, and social covariates. Within individuals, increases in alcohol consumption relative to one’s own average were associated with lower concurrent life satisfaction, with this pattern seen primarily among men. Conversely, improvements in nutrition and sleep were associated with a negligible higher concurrent life satisfaction in the aggregated and gender-stratified models. Higher average physical activity, better nutrition and sleep, and lower average sedentary time were all associated with higher life satisfaction, with only nutrition showing an appreciable effect size. Cross-sectional bivariate analyses showed significant associations between all health behaviours and life satisfaction at each wave, including a positive association between alcohol frequency and life satisfaction.

Our findings showed that life satisfaction declined marginally after accounting for health behaviours and other covariates, aligning with the WHO’s [8] Healthy Ageing Framework, which emphasises that well-being in later life reflects the interplay among intrinsic capacity, health behaviours, and environmental supports. Although women reported slightly lower life satisfaction than men, the difference was trivial in magnitude. Women’s slightly lower life satisfaction may reflect longer exposure to poorer health outcomes over the life course [39], as well as broader gender inequalities and role-related factors, including lower socioeconomic resources and greater cumulative caregiving responsibilities [16, 17].

The bivariate analyses demonstrated that higher alcohol frequency was significantly associated with higher life satisfaction at each wave, suggesting that alcohol use in older adulthood may reflect social engagement [40]. However, the growth-curve model showed that increases in alcohol use over time within individuals were linked to declines in life satisfaction. This apparent contradiction underscores the importance of distinguishing between-person differences from within-person change. While those who drink more on average may report higher life satisfaction due to social or contextual advantages [40], increases in alcohol consumption within individuals over time may reflect a maladaptive coping strategy for psychosocial stress rather than recreational use [18, 19]. Gender-stratified analyses indicated that this effect was significant in men but not in women, with the observed difference approaching or exceeding a plausible minimally important difference on the SWLS, reflecting gendered norms around drinking. Canadian men tend to drink more regularly and heavily than women [41], and increases in alcohol use among men may represent reactive drinking in response to stressors [18, 19], contributing to faster declines in life satisfaction through health deterioration [42]. By contrast, women’s alcohol use is more often embedded in social contexts and less closely linked to stress reactivity [18, 19]. Moreover, women are more likely to employ alternative coping strategies, such as seeking social support or professional help [43], instead of risky alcohol use, which may serve as a protective mechanism that reduces the rate of life satisfaction decline [44].

The association observed for between-person nutrition suggests that individuals with generally healthier diets tend to report meaningfully higher life satisfaction than their peers, with differences approaching or exceeding a plausible minimally important difference on the SWLS. In contrast, within-person variation in nutrition appeared to yield smaller effects, indicating that stable dietary differences across individuals may be more consequential for subjective well-being than short-term fluctuations. A stable, healthy diet supports physiological functioning, boosts energy levels, reduces the risk of chronic disease, and enhances body image, self-esteem, and mood [45, 46], all of which are foundational to positive life evaluations. By comparison, sleep showed a consistently small effect size in both within- and between-person level associations. Nonetheless, adequate sleep is essential for the body’s restorative processes, especially among older adults, to reduce the rate of age-related decline [47] and to support mood regulation and cognitive functioning [13, 48], thereby contributing to higher life satisfaction. The effect of sleep on life satisfaction appears relatively proximal [49], whereas the benefits of other health behaviours, such as physical activity and sedentary time, may accumulate gradually over time.

At the between-person level, older adults who were consistently more physically active, less sedentary, and who obtained adequate sleep reported slightly higher life satisfaction, consistent with evidence that balanced daily movement patterns support health outcomes and well-being [50, 51]. These behaviours function as an interdependent continuum, in which time spent in one activity displaces time from another [50, 51]. The Canadian 24-Hour Movement Guidelines for Older Adults operationalise this concept by integrating sleep, sedentary behaviour, and physical activity across the full day [52]. The guidelines recommend 7–8 h of sleep, no more than 8 h of sedentary time (including a maximum of 3 h of recreational screen time), and the remainder allocated to light, moderate, or vigorous physical activity. The stronger associations observed at the between-person level for physical activity and sedentary time, compared with within-person changes, may indicate that older adults who consistently adhered to these guidelines over time experienced higher life satisfaction than their peers, whereas short-term fluctuations had a limited impact.

Importantly, physical activity demonstrated a gender-specific pattern: men with higher average physical activity reported slightly greater life satisfaction, whereas this association was not observed among women. For older men, sustained physical activity supports functional capacity, personal identity, and enjoyment, thereby enhancing life satisfaction [53, 54]. In contrast, older women typically engage in less long-term physical activity [55], which may explain the absence of a comparable effect on their life satisfaction.

Although most statistically significant associations in this study had small effect sizes, they may still be meaningful at the population level. Health behaviours are modifiable, and even small improvements in life satisfaction associated with them can translate into substantial population gains, consistent with Rose’s population strategy, which emphasises that small shifts in risk or well-being across large populations can yield important public health benefits [56]. In general, most associations between health behaviours and life satisfaction were directionally similar for men and women and differed only modestly in magnitude, suggesting that population-level interventions do not require substantial gender differentiation, as the same core determinants appear relevant across groups. However, an important exception was observed for within-person changes in alcohol use, where the association with reduced life satisfaction was more pronounced among men than women, indicating the potential value of gender-sensitive alcohol-related health promotion strategies in older adults.

## Implications of findings

The outcomes of this study will benefit ageing research, clinical practice, policy development, and individual decision-making. The findings provide longitudinal, population-based evidence on the influence of health behaviours on life satisfaction, addressing a critical gap in the literature, which has been dominated by cross-sectional designs. The use of within-person and between-person analyses highlights that both changes in behaviours and long-term individual differences are associated with life satisfaction in later life.

The study findings have direct relevance for clinical practice and the delivery of geriatric care. Since poor life satisfaction is linked to disability, morbidity, and mortality in later life [6, 7], its decline may signal emerging health vulnerabilities before overt clinical deterioration occurs. Routine assessment of life satisfaction using brief single-item or validated scales during primary care or geriatric visits may serve as a low-cost screening tool to identify older adults at risk of functional decline.

Although promoting healthy behaviours is embedded in clinical practice to prevent disease, our findings indicate that these behaviours are relevant for well-being. Evidence that changes in health behaviours are associated with concurrent changes in life satisfaction underscores healthy ageing as a dynamic process requiring ongoing clinical monitoring rather than a one-time lifestyle intervention. We recommend that repeated assessments of alcohol use, nutrition, sleep, physical activity, and sedentary time be included in routine care, as they may help clinicians detect early deterioration and commence immediate intervention. For example, rising alcohol use, particularly among men, may prompt motivational interviewing [57] or referral to community alcohol reduction programmes; declines in nutrition or sleep may trigger dietitian referral, community meal programmes, or cognitive behavioural therapy for insomnia; and sustained physical activity should be reinforced through physiotherapy-guided prescriptions and community exercise programmes. A holistic intervention approach that aligns with the Canadian 24-Hour Movement Guidelines (i.e., more movement, less sedentary time, and adequate, high-quality sleep) should be prioritised rather than focusing on single behaviours in isolation.

At the policy level, these findings provide empirical support for the mandate of the CIHR Institute of Aging [4], which seeks to promote healthy ageing and enhance quality of life in older adulthood. They strengthen the case for sustained public health investment across all levels of government in population-level strategies that promote healthy behaviours and support well-being in later life. Given that nutrition showed the most meaningful association with life satisfaction, greater policy commitment to nutrition interventions, such as front-of-package nutrition labelling and initiatives that improve the affordability and accessibility of healthy foods, may support healthier dietary patterns and improve well-being among older adults. Similarly, given the stronger association between increased alcohol use and lower life satisfaction, particularly among men, strengthening existing alcohol control measures, including federal excise taxation, provincial pricing and retail controls, and impaired driving regulations, alongside gender-sensitive prevention strategies such as targeted public health campaigns with messaging tailored to older men, may help reduce harmful alcohol use and support well-being in older adulthood.

Health behaviours are modifiable and largely within individual control [11]. Our findings underscore the potential for individuals to shape their own well-being across the life course. Individuals are encouraged to adopt and maintain health-promoting behaviours early in adulthood to enhance satisfaction in older age. Following the Canadian 24-Hour Movement Guidelines offers a practical framework for balancing physical activity, sedentary behaviour, and sleep to enhance and maintain well-being over time.

## Strengths and limitations

A key strength of this study is the use of linear mixed-effects growth curve modelling, which is well-suited to longitudinal data. This approach allowed us to retain participants with incomplete follow-up data due to mortality or loss to follow-up, reducing bias associated with listwise deletion. Second, the models were adjusted for sociodemographic, health, and social covariates, reducing potential confounding and enabling a more precise estimation of the longitudinal associations between health behaviours and life satisfaction.

The limitations include the use of questionnaires for data collection, which is prone to self-report bias, particularly recall and social desirability biases. The study is observational; hence, causal inferences cannot be drawn from the results. The CLSA data collection was conducted predominantly in urban areas across seven of the ten Canadian provinces and required a minimum level of proficiency to be eligible [22], which may have underrepresented rural-dwelling and less literate older adults. Furthermore, the mixed-effects models were estimated using the lme4 package in R, which accounts for within-person clustering across repeated waves through the random-effects structure but does not incorporate the stratified, clustered survey design into variance estimation. The weighted baseline of CLSA was comparable across many variables with estimates from other nationally representative surveys and addresses unequal probabilities of selection [22], but may not, by itself, yield design-correct standard errors. Readers should therefore interpret statistically significant findings alongside the effect sizes and predicted differences reported for each estimate, which are unaffected by this limitation.

## Conclusions

This study provides evidence on how changes in health behaviours relate to trajectories of life satisfaction among older Canadian adults, with gender distinctions. Women reported very slightly lower life satisfaction, but men experienced a marginal decline over time. Better nutrition and sufficient sleep were associated with greater life satisfaction, while increasing alcohol use is linked to lower life satisfaction among men. Sustained higher physical activity in men and lower sedentary time were associated with greater life satisfaction. The findings demonstrate that changes in health behaviours and the maintenance of healthy habits are linked with differences in older adults’ life satisfaction. Monitoring health behaviours alongside life satisfaction allows policymakers and healthcare professionals to spot early declines and provide timely, tailored support. For individuals, maintaining health-promoting behaviours across the life course could improve life satisfaction in later life.

## Data Availability

Data are available from the Canadian Longitudinal Study on Aging (www.clsa-elcv.ca) for researchers who meet the criteria for access to de-identified CLSA data.

## Abbreviations

AB SCREEN™ II: Abbreviated Seniors in the Community Risk Evaluation for Eating and Nutrition II
CIHR: Canadian Institutes of Health Research
CLSA: Canadian Longitudinal Study on Aging
Pd: Predicted difference
SD: Standard deviation
SPSS: Statistical Package for the Social Sciences
SWLS: Satisfaction with Life Scale
WHO: World Health Organisation
